# Duvalier Regime in Haiti and Immigrant Health in the United States

**DOI:** 10.29024/aogh.2366

**Published:** 2018-11-05

**Authors:** Jeremy C. Green, Amanda Schoening, Michael G. Vaughn

**Affiliations:** 1 Saint Louis University, Department of Health Management and Policy, US; 2 Saint Louis University, School of Social Work, US

## Abstract

**Background:**

Haitians immigrate to the United States for many reasons, including the opportunity to escape political violence. The extant literature on Haitian immigrant health focuses on post-migration, rather than pre-migration, environments and experiences.

**Objective:**

In this study, we analyze health outcomes data from a nationally representative sample of Haitian immigrants in the United States from 1996 to 2015. We estimate age-adjusted associations between pre-migration residence in Haiti during the repressive regimes and generalized terror of Francois and Jean-Claude Duvalier, who ran Haiti from 1957 to 1986.

**Methods:**

We used ordered probit regression models to quantify age-adjusted associations between the duration of pre-migration residence in Haiti during the Duvalier regime, and the distribution of post-migration health status among Haitian immigrants in the United States.

**Findings:**

Our study sample included 2,438 males and 2,800 females ages 15 and above. The mean age of males was 43.5 (standard deviation, 15.5) and the mean age of females was 44.7 (standard deviation, 16.6). Each additional decade of pre-migration residence in Haiti during the Duvalier regime is associated with a 2.9 percentage point decrease (95% confidence interval 0.6 to 5.3) in excellent post-migration health for males, and a 2.8 percentage point decrease (95% confidence interval, 0.8 to 4.8) for females. Within the subsample of Haitian immigrants with any pre-migration residence in Haiti during the Duvalier regime, each additional decade since the regime is associated with a 3.3 percentage point increase (95% confidence interval, 1.2 to 5.5) in excellent post-migration health for males, and a 2.3 percentage point increase (95% confidence interval, 0.5 to 4.1) for females.

**Conclusions:**

Overall, we found statistically significant and negative associations between the Duvalier regime and the post-migration distribution of health status 10 to 57 years later. We found statistically significant and positive associations between the length of time since the Duvalier regime and post-migration health.

More individuals immigrate to the United States from Haiti than from any other country in the Caribbean [1] and there are over half a million Haitian immigrants in the United States [2]. Unfortunately, however, there is little research on the health of Haitian immigrants [3,4]. Most public health studies compare black and white individuals, without examining Haitian immigrants as a distinct community with their own culture and history [5]. In fact, many Haitian immigrants identify with cultural, linguistic, and social aspects of their heritage, rather than identifying with a race [6]. Public health research on Haitian immigrants can be limited for a number of reasons including a lack of trust in individuals outside of their community, a lack of confidence in scientific research [7], cultural practices that conflict with Western medicine [8,9,10,11,12], and stigmas associated with health conditions [13].

Individuals have migrated from Haiti to the United States since the early in the nineteenth century, but Haitian immigrants continue to lag behind other immigrants in terms of their health and economic outcomes [14]. The number of immigrants from Haiti to the United States increased dramatically in the 1970s and 1980s, as many Haitians fled persecution and poverty in their homeland [15]. Haiti is one of the poorest countries in the world, and United States refugee policy is considered to be biased against Haitian refugees [16]. Although the United States government does not consider Haitian immigrants to be refugees solely on the basis of their country of birth, Haitian immigrants have similar outcomes as refugees in the United States because they come from an environment of insecurity, political violence, and poverty [17]. Haitian immigrants also come from rural areas [18], lack education [19] and health insurance coverage [20,21], and suffer from an underuse of preventive health care services as compared other Caribbean immigrants [22,23,24,25].

Francois Duvalier was president of Haiti from 1957 to his death in 1971, when his son, Jean-Claude Duvalier became president [26]. The Duvalier regime interrupted what was otherwise a prolonged period of relative political stability in Haiti [27]. Even when compared to other dictators, Francois Duvalier is considered especially narcissistic and sadistic [28], and Haiti under Duvalier was a place of chaos, repression, terror, trauma, and murder [29,30]. Francois Duvalier militarized his regime and created the Volunteers for National Security, or *Tons Tons Macoutes*, a paramilitary organization that Francois Duvalier used to support his regime and its power [31]. Many individuals left Haiti in response to the political violence associated with the Duvalier regime [32,33].

Exposure to pre-migration political violence can have important consequences for the health status of immigrants in their new countries. Rousseau and Drapeau [34] surveyed immigrants from several countries, including Haiti, to Quebec, Canada to study their pre-migration experiences of political violence in relation to their post-migration mental health outcomes. The study found disturbing levels of exposure to political violence and related traumatic experience in Haiti. Among Haitian immigrants in the study, 56% migrated from Haiti to North America for political reasons, 46% witnessed violent acts or persecution in Haiti, and 27% experienced persecution of themselves or their family members in Haiti. Witnessing violent acts of persecution in Haiti was associated with statistically significant increases in post-migration emotional stress measures that include anxiety and depression. Haitian immigrants also suffer post-migration depression and post-traumatic stress disorder [35]. Fawzi et al. [17: p482] collected survey data on 164 students in Boston public high schools who immigrated to the United States from Haiti. The study found that in this population the prevalence of depression was 12%, and the prevalence of post-traumatic stress disorder was 14% – rates that are comparable to refugee populations. Despite the comparatively high prevalence of depression and post-traumatic stress disorder among Haitian immigrants, Haitians and other Caribbean immigrants suffer from an underutilization of formal mental health care services [36].

Despite the problematic pre-migration experiences and disappointing health status of Haitian immigrants, we are unaware of much research that quantifies concrete pre-migration political environments in relation to post-migration health outcomes. Our research takes advantage of a unique data source to evaluate the health status of Haitian immigrants to the United States, in relation to the number of years of pre-migration residence in Haiti during the Duvalier regime, during or after their year of birth and before or during their year of migration from Haiti to the United States. Our working hypothesis is that the duration of pre-migration residence in Haiti during the Duvalier regime is negatively associated with the distribution of post-migration health status of Haitian Americans. We further hypothesized that the associations between the Duvalier regime and post-migration health will fade over time, and that the length of time since the Duvalier regime will be positively associated with the distributions of post-migration health status.

## Methods

We analyzed data from a nationally representative sample of Haitian Americans to estimate associations between pre-migration residence in Haiti during the Duvalier regime and the post-migration distribution of health status among Haitian Americans in the United States. We used regression analysis to adjust the estimated associations between pre-migration residence in Haiti during the Duvalier regime and the post-migration outcomes for the observed distributions of age trends, and reported results stratified by gender. In the following sections, we provide some more details about our study dataset, review the coding of our study variables, and describe our methods of statistical analysis.

### Study Dataset

We analyzed data from the Integrated Public Use Microdata Series, Current Population Survey and Annual Social and Economic Supplement [37]. The Current Population Survey contains data on the birthplace of each individual surveyed and the Annual Social and Economic Supplement contains data on their self-rated health status. The surveys are fielded each March by the United States Bureau of Labor Statistics and the United States Census Bureau. Data are collected through in-person and telephone interviews from a nationally representative sample of the non-institutionalized population in the United States. The study dataset is underutilized in the population health literature on immigrant comparisons. Researchers have used this information to study the health of Caribbean immigrants in general, but not Haitian immigrants in particular [38]. While the dataset does contain information on self-rated health status and other outcome measures including household income and educational attainment, the dataset is limited by its lack of information on some other variables that might be relevant to population health studies of political turmoil, such as family history, and mental health.

The Current Population Survey supplements, including the Annual Social and Economic Supplement, contain probability weights. The inverse probability weights help to adjust analyses for non-response and other aspects of the survey, by weighting results by the inverse probability of inclusion in the study sample, which can help to generate estimates that are comparable to the non-institutionalized population of the United States. For the study sample, we selected individuals who were born in Haiti and who were above the age of 15 at the time of the surveys from 1996 through 2015.

### Study Variables

For the dependent variable, we used the distribution of self-rated health status on the Likert scale of excellent health, very good health, good health, fair health, or poor health. For the key independent variable, we used the continuous, linear trend in number of decades of pre-migration residence in Haiti during either the Francois Duvalier regime or the Jean-Claude Duvalier regime. For each year of the Duvalier regime, an individual was considered exposed to the regime if they were born before or during a given year and migrated to the United States during or after a given year. We included years 1957—1971, when Francois Duvalier was in power, and years 1972—1986 when Jean-Claude Duvalier was in power. For each individual included in the study sample, we counted the number of decades of pre-migration residence in Haiti during the Duvalier regime before migration to the United States.

To test the hypothesis that estimated associations between pre-migration residence in Haiti during the Duvalier regime and the post-migration distribution of health status faded over time, we replaced the key independent variable of decades of pre-migration residence during the Duvalier regime with a secondary key independent variable for the number of decades since the most recent year of pre-migration residence in Haiti during the Duvalier regime. For the analysis of the number of decades since the Duvalier regime, we included in the sample only those individuals with at least one year of pre-migration residence in Haiti during the Duvalier regime.

In addition to the main analysis of self-rated health, we also examined relationships between pre-migration residence in Haiti during the Duvalier regime, and the post-migration distributions of income and education among Haitian immigrants in the United States. During the Duvalier regime, Haitians suffered from an inadequate education system [39] and widespread poverty [40]. As a secondary aim of our study, we test the hypothesis that the associations of the Duvalier regime with household income and educational attainment persisted over a longer period of time and could be detected among Haitian immigrants in the United States. As our study sample included individuals ages 15 and above, we created a dichotomous variable to identify those individuals in our study sample who had completed any high school education by the time of the survey, as compared those individuals with less than a high school education at the time of the survey. For our measure of household income, we used the categories of household income as a percent of the federal poverty level, as defined in the Current Population Survey. The federal poverty level is defined by the United States Department of Health and Human Surveys by comparing a family’s income to a poverty threshold that varies based on the number of individuals in the family. Families with greater numbers of children require greater income to exceed the federal poverty level for their family size in a given year.

### Statistical Analysis

To estimate associations of years of exposure to the Duvalier regime in Haiti, with health and related outcomes in the United States, we fit ordered probit regression models [41]. Covariates were included in the model to adjust for continuous trends in linear age, age squared, and age cubed. These continuous, polynomial age adjustments, referred to as the fractional polynomial method in the medical literature [42], can help to remove the residual confounding that can otherwise result from nonlinear relationships between age and health. To demonstrate the robustness of the age adjustments to differences in the order of polynomials, we also reported results from models adjusted for quadratic age trends [42: p403]. After fitting the regression models, we predicted the average marginal effects of each additional year of exposure to the Duvalier regime, on the distribution of self-rated health outcomes, the probability of having completed any high school education by the time of the survey, and the probability of living in a family with income above the federal poverty level. We used the delta method to estimate 95% confidence intervals for the associations between pre-migration residence in Haiti during the Duvalier regime and post-migration health status [43]. Estimated associations and 95% confidence intervals were computed using the survey sampling weights. We used Stata statistical software, version 14, for data analysis [44].

## Results

Our study sample included 2,438 males and 2,800 females ages 15 and above. The mean age of males was 43.5 (standard deviation, 15.5) and the mean age of females was 44.7 (standard deviation, 16.6). We report the results from our study in Table 1, 2, 3. Table 1 contains the descriptive results. Tables 2 and 3 contain the regression results.

**Table 1:** Health of Haitian Immigrants, by Pre-Migration Exposure to the Duvalier Regime^a^.

Health	Pre-migration residence in Haiti during the Duvalier regime,^b^ No. (%)	No pre-migration residence in Haiti during the Duvalier regime, No. (%)	*P* Value^c^

**Males (n = 2 438)**			

Excellent	545 (24.27)	70 (36.46)	<.001
Very good	759 (33.79)	72 (37.50)	
Good	686 (30.54)	43 (22.40)	
Fair	194 (8.638)	4 (2.083)	
Poor	62 (2.760)	3 (1.562)	
Total	2246 (100)	192 (100)	
**Females (n = 2 800)**			

Excellent	552 (21.21)	79 (39.90)	<.001
Very good	818 (31.44)	67 (33.84)	
Good	824 (31.67)	41 (20.71)	
Fair	278 (10.68)	6 (3.030)	
Poor	130 (4.996)	5 (2.525)	
Total	2602 (100)	198 (100)	

^a^ Table entries are counts and frequencies of Haitian immigrants in the United States. ^b^ Individuals who were born in Haiti before or during the Duvalier regime and migrated to the United States during or after the Duvalier regime. ^c^ *P* values are from chi-square tests.

**Table 2:** Decades of the Duvalier Regime in Haiti and Immigrant Health in the United States^a^.

Health status	Average Marginal Effect (95% Confidence Interval)^b^	

Gender	Males (n = 2 438)	Females (n = 2 800)

	**Unadjusted**	

Excellent	–0.0731 [–0.0884, –0.0577]	–0.0846 [–0.0980, –0.0712]
Very good	–0.0178 [–0.0227, –0.0128]	–0.0281 [–0.0337, –0.0226]
Good	0.0482 [0.0379, 0.0584]	0.0496 [0.0415, 0.0577]
Fair	0.0293 [0.0222, 0.0364]	0.0356 [0.0290, 0.0422]
Poor	0.0134 [0.00936, 0.0174]	0.0275 [0.0216, 0.0335]
	**Adjusted**^c^	

Excellent	–0.0293 [–0.0529, –0.00582]	–0.0280 [–0.0481, –0.00786]
Very good	–0.00680 [–0.0123, –0.00130]	–0.00864 [–0.0149, –0.00238]
Good	0.0194 [0.00381, 0.0350]	0.0166 [0.00461, 0.0286]
Fair	0.0116 [0.00226, 0.0209]	0.0114 [0.00320, 0.0197]
Poor	0.00519 [0.000959, 0.00943]	0.00859 [0.00231, 0.0149]

^a^ Table entries are average marginal effects and 95% confidence intervals from ordered probit regressions of the distribution of health status on the number of decades of pre-migration residence in Haiti during the Duvalier regime. For each individual, the number of decades of pre-migration residence in Haiti during the Duvalier regime includes those years of the Duvalier regime during or after their year of birth and before or during their year of migration. ^b^ Estimates and 95% confidence intervals are weighted by the sampling probabilities. ^c^ Adjustments are for the observed distributions of age, age squared, and age cubed.

**Table 3:** Decades since the Duvalier Regime in Haiti and Immigrant Health in the United States^a^.

Health status	Average Marginal Effect (95% Confidence Interval)^b^	

Gender	Males (n = 2 246)	Females (n = 2 602)

	**Unadjusted**	

Excellent	–0.0290 [–0.0476, –0.0104]	–0.0463 [–0.0623, –0.0302]
Very good	–0.00795 [–0.0133, –0.00256]	–0.0182 [–0.0249, –0.0114]
Good	0.0191 [0.00685, 0.0314]	0.0273 [0.0179, 0.0367]
Fair	0.0123 [0.00422, 0.0204]	0.0212 [0.0134, 0.0289]
Poor	0.00553 [0.00174, 0.00933]	0.0160 [0.00976, 0.0221]
	**Adjusted**^c^	

Excellent	0.0331 [0.0116, 0.0546]	0.0234 [0.00554, 0.0413]
Very good	0.00875 [0.00299, 0.0145]	0.00870 [0.00205, 0.0153]
Good	–0.0219 [–0.0361, –0.00769]	–0.0140 [–0.0248, –0.00326]
Fair	–0.0139 [–0.0229, –0.00477]	–0.0104 [–0.0184, –0.00242]
Poor	–0.00612 [–0.0103, –0.00197]	–0.00769 [–0.0136, –0.00180]

^a^ Table entries are average marginal effects and 95% confidence intervals from ordered probit regressions of the distribution of health status on the number of decades since pre-migration residence in Haiti during the Duvalier regime. For each individual, pre-migration residence in Haiti during the Duvalier regime includes those years of the Duvalier regime during or after their year of birth and before or during their year of migration from Haiti to the United States. ^b^ Estimates and 95% confidence intervals are weighted by the sampling probabilities. ^c^ Adjustments are for the observed distributions of age, age squared, and age cubed.

In Table 1, we report the distribution of post-migration health status for Haitian immigrants to the United States in the study sample by pre-migration residence in Haiti during the time period of the Duvalier regimes. For males and females, we found statistically significant differences in the distribution of post-migration health status, by pre-migration residence in Haiti during the Duvalier regime. Overall, Haitian immigrants in the United States who resided in Haiti during the Duvalier regime reported poor post-migration health status, as compared to those who did not reside in Haiti during the Duvalier regime. Among males who resided in Haiti during the Duvalier regime, 24.3% reported excellent post-migration health as compared to 36.5% of males who did not reside in Haiti during the Duvalier regime. Similarly, 21.2% of females who resided in Haiti during the Duvalier regime reported excellent post-migration health, as compared to 39.9% of females who did not reside in Haiti during the Duvalier regime.

In Table 2, we report results from the regression analysis of associations between post-migration health status and the duration of pre-migration residence in Haiti during the time period of the Duvalier regime. The first column of Table 2 repeats the labels on the categories of post-migration health status from Table 1. The second and third columns of Table 2 report average marginal effects, and corresponding 95% confidence intervals, on the associations between post-migration health status and the number of decades of pre-migration residence in Haiti during the Duvalier regime. The second column of Table 2 reports the results for males; the third column of Table 2 reports the results for females. After including the age adjustments in the analysis, we found statistically significant and negative associations between the duration of pre-migration residence in Haiti during the time period of the Duvalier regime, and the post-migration distribution of health status in the United States. Each additional decade of pre-migration residence in Haiti during the Duvalier regime is associated with a 2.9 percentage point decrease (95% confidence interval, 0.6 to 5.3) in the post-migration probability of excellent health in the United States for males, and a 2.8 percentage point decrease (95% confidence interval, 0.8 to 4.8) for females.

In Table 3, we report the results from a regression analysis similar to that from Table 2. In Table 3, we restrict the analysis to the subsample of individuals in the study sample with any duration of pre-migration residence in Haiti during the Duvalier regime, and replace the dependent variable with the number of decades since the most recent year of pre-migration residence in Haiti during the time period of the Duvalier regime. After adjusting the estimated associations for the observed distributions of age, age squared, and age cubed, we found statistically significant and positive associations between the number of decades since pre-migration residence in Haiti during the Duvalier regime, and the post-migration distribution of health status among Haitian immigrants in the United States. Each additional decade since pre-migration residence in Haiti during the Duvalier regime was associated with a 3.3 percentage point increase (95% confidence interval, 1.2 to 5.5) in the probability of excellent post-migration health status for males, and a 2.3 percentage point increase (95% confidence interval, 0.6 to 4.1) for females.

In Table 4, we report our findings on age-adjusted associations between pre-migration residence in Haiti during the Duvalier regime and the post-migration distributions of educational attainment and household income. Similar to our findings on health, we find negative and statistically significant age-adjusted associations of the number of decades of pre-migration residence in Haiti during the Duvalier regime, with the post-migration probabilities of having any high school education and living in a household with income above the federal poverty level. Similar to our findings on health, we find positive and statistically significant associations of the number of decades since the most recent year of pre-migration residence in Haiti during the Duvalier regime, with completing any high school education and living in a household with income above the federal poverty level, among the sample of individuals with at least one year of pre-migration residence in Haiti during the Duvalier regime.

**Table 4:** Duvalier Regime in Haiti and Immigrant Education and Income in the United States^a^.

	Average Marginal Effect (95% Confidence Interval)^b^	

	**Decades of the Duvalier regime**	

	**Males (n = 2 438)**	**Females (n = 2 800)**

Education^c^	–0.0800 [–0.107, –0.0527]	–0.141 [–0.164, –0.117]
Income^d^		
150% FPL and above	–0.102 [–0.135, –0.0687]	–0.117 [–0.148, –0.0872]
125 to 149% FPL	0.0138 [0.00881, 0.0188]	0.00923 [0.00633, 0.0121]
100 to 124% FPL	0.0136 [0.00850, 0.0186]	0.0167 [0.0118, 0.0217]
Below 100% FPL	0.0745 [0.0498, 0.0991]	0.0915 [0.0675, 0.116]
	**Decades since the Duvalier regime**	

	**Males (n = 2 246)**	**Females (n = 2 602)**

Education	0.0839 [0.0596, 0.108]	0.112 [0.0933, 0.130]
Income		
150% FPL and above	0.0773 [0.0457, 0.109]	0.115 [0.0869, 0.142]
125 to 149% FPL	–0.0112 [–0.0162, –0.00626]	–0.00889 [–0.0116, –0.00615]
100 to 124% FPL	–0.0106 [–0.0154, –0.00579]	–0.0171 [–0.0220, –0.0123]
Below 100% FPL	–0.0555 [–0.0783, –0.0326]	–0.0886 [–0.111, –0.0667]

FPL = federal poverty level. ^a^ Table entries are average marginal effects and 95% confidence intervals from ordered probit regressions of the distribution of the outcomes on the number of decades of the Duvalier regime, and the number of decades since pre-migration residence during the Duvalier regime, adjusted for the observed distributions of cubic age trends. ^b^ Estimates and 95% confidence intervals are weighted by the sampling probabilities. ^c^ Educational attainment identifies those individuals who completed any high school education. ^d^ Household income categories compare total family income given the number of children in the family, to poverty thresholds defined annually by the United States Department of Health and Human Services. Households with income below 100% of the FPL live in poverty.

In Table 5, we combine our findings on age-adjusted associations between pre-migration residence in Haiti during the Duvalier regime and the post-migration distributions of health status, educational attainment, and household income in our nationally representative sample of Haitian immigrants in the United States. In these results, we add quadratic age trends to allow additional for additional nonlinearities in the relationships between age and health. Overall, for both key independent variables of the number of decades of pre-migration residence in Haiti during the Duvalier regime, and the number of decades since pre-migration residence in Haiti during the Duvalier regime, our findings from regressions specified with quadratic age trends are similar to the findings presented in Tables 2, 3, 4 from regressions with cubic age trends. These findings help to demonstrate the robustness of our statistical analysis to varying degrees of polynomials in the continuous age trend adjustments.

**Table 5:** Robustness of Associations of the Duvalier Regime in Haiti with Immigrant Health, Education, and Income in the United States to Quadratic Age Trend Adjustments^a^.

	Average Marginal Effect (95% Confidence Interval)^b^	

	**Decades of the Duvalier regime**	

**Health status**	**Males (n = 2 438)**	**Females (n = 2 800)**

Excellent	–0.0314 [–0.0555, –0.00722]	–0.0310 [–0.0514, –0.0105]
Very good	–0.00725 [–0.0129, –0.00159]	–0.00949 [–0.0158, –0.00317]
Good	0.0207 [0.00474, 0.0367]	0.0184 [0.00618, 0.0306]
Fair	0.0124 [0.00278, 0.0219]	0.0126 [0.00425, 0.0209]
Poor	0.00555 [0.00118, 0.00992]	0.00948 [0.00311, 0.0158]
Education^c^	–0.0774 [–0.105, –0.0497]	–0.143 [–0.167, –0.120]
Income^d^		
150% FPL and above	–0.0988 [–0.133, –0.0646]	–0.127 [–0.158, –0.0969]
125 to 149% FPL	0.0134 [0.00835, 0.0184]	0.00997 [0.00700, 0.0129]
100 to 124% FPL	0.0132 [0.00805, 0.0183]	0.0181 [0.0131, 0.0232]
Below 100% FPL	0.0722 [0.0468, 0.0976]	0.0992 [0.0750, 0.123]
	**Decades since the Duvalier regime**	

**Health status**	**Males (n = 2 246)**	**Females (n = 2 602)**

Excellent	0.0326 [0.0110, 0.0542]	0.0241 [0.00613, 0.0420]
Very good	0.00863 [0.00284, 0.0144]	0.00890 [0.00226, 0.0155]
Good	–0.0216 [–0.0358, –0.00731]	–0.0144 [–0.0253, –0.00361]
Fair	–0.0136 [–0.0228, –0.00453]	–0.0106 [–0.0186, –0.00266]
Poor	–0.00602 [–0.0102, –0.00187]	–0.00789 [–0.0138, –0.00199]
Education	0.0835 [0.0592, 0.108]	0.112 [0.0935, 0.130]
Income		
150% FPL and above	0.0750 [0.0433, 0.107]	0.116 [0.0887, 0.144]
125 to 149% FPL	–0.0109 [–0.0159, –0.00597]	–0.00900 [–0.0117, –0.00626]
100 to 124% FPL	–0.0103 [–0.0151, –0.00550]	–0.0174 [–0.0222, –0.0125]
Below 100% FPL	–0.0537 [–0.0767, –0.0308]	–0.0899 [–0.112, –0.0682]

FPL = federal poverty level ^a^ Table entries are average marginal effects and 95% confidence intervals from ordered probit regressions of the distribution of the outcomes on the number of decades of the Duvalier regime, and the number of decades since pre-migration residence during the Duvalier regime, adjusted for the observed distributions of quadratic age trends. ^b^ Estimates and 95% confidence intervals are weighted by the sampling probabilities. ^c^ Educational attainment identifies those individuals who completed any high school education. ^d^ Household income categories compare total family income given the number of children in the family, to poverty thresholds defined annually by the United States Department of Health and Human Services. Households with income below 100% of the FPL live in poverty.

## Discussion

Results from our study highlight the importance of pre-migration political environments in quantifying the post-migration health outcomes of Haitian immigrants to the United States. In the case of pre-migration residence in Haiti during the Duvalier regime, we found that effects of the pre-migration regime on post-migration health outcomes were additive and cumulative in the number of years of pre-migration residence in Haiti during the Duvalier regime. Our findings are consistent with the overall results of earlier studies that find high rates of pre-migration political violence and high rates of post-migration health problems in Haitian American populations [[Bibr B17][Bibr B34]]. Our findings on the long-term, transnational consequences extend the literature describing short-term, local relationships between the Duvalier regime, the education system, and poverty in Haiti. Researchers should further explore the mechanisms through which the Duvalier regime is associated with decreases in the probability of excellent self-rated health status among Haitian immigrants in the United States, and how effects of the regime on health, education, and income may relate to and interact with one another.

Strengths of our study include its use of a quantitative measure of the pre-migration environment, which in our experience is rare in the literature on immigrant health comparisons. Another asset of our study is its measurement of long-term health effects of the Duvalier regime, as we measured the distribution of health status 10 to 57 years after the most recent year of pre-migration residence in Haiti during the Duvalier regime. Our study dataset is itself a strength of our research, as we are unaware of many if any other datasets that include data from such large, nationally representative samples of immigrants from a single country of birth collected over an extended time period. This paper demonstrates the types of unique research questions that can be answered concerning the pre-migration environment and its influence on post-migration health and related outcomes. Limitations of our study include lack of data on the complete migration history of individuals in the study, who might have lived in different countries after being born in Haiti and before migrating to the United States. More research is needed to understand the mechanisms through which the Duvalier regime can impact health, for example whether the regime effects are mostly economic, and if there are psychological effects via generalized perceived terror. Results of the study are specific to the experience of Haitian immigrants and to the Duvalier regime, and might not generalize to immigrants from other countries, or to their experiences with other dictators.

Future research is needed on the reasons that individuals migrate from Haiti to the United States, to better separate out the migration experience from the pre-migration environment. More data is needed on the migration experience of Haitian immigrants to the US, including data on Haitians who attempted to migrate and were returned to Haiti by the United States government, to compare the outcomes of individuals who managed to migrate from Haiti to the United States, to those who were forcibly returned to Haiti.
